# Polyketide Synthase and Nonribosomal Peptide Synthetase Gene Clusters in Type Strains of the Genus *Phytohabitans*

**DOI:** 10.3390/life10110257

**Published:** 2020-10-27

**Authors:** Hisayuki Komaki, Tomohiko Tamura

**Affiliations:** Biological Resource Center, National Institute of Technology and Evaluation (NBRC), Chiba 292-0818, Japan; tamura-tomohiko@nite.go.jp

**Keywords:** actinomycete, genome, *Phytohabitans*, polyketide, nonribosomal peptide

## Abstract

(1) Background: *Phytohabitans* is a recently established genus belonging to rare actinomycetes. It has been unclear if its members have the capacity to synthesize diverse secondary metabolites. Polyketide and nonribosomal peptide compounds are major secondary metabolites in actinomycetes and expected as a potential source for novel pharmaceuticals. (2) Methods: Whole genomes of *Phytohabitans flavus* NBRC 107702^T^, *Phytohabitans rumicis* NBRC 108638^T^, *Phytohabitans houttuyneae* NBRC 108639^T^, and *Phytohabitans suffuscus* NBRC 105367^T^ were sequenced by PacBio. Polyketide synthase (PKS) and nonribosomal peptide synthetase (NRPS) gene clusters were bioinformatically analyzed in the genome sequences. (3) Results: These four strains harbored 10, 14, 18 and 14 PKS and NRPS gene clusters, respectively. Most of the gene clusters were annotated to synthesis unknown chemistries. (4) Conclusions: Members of the genus *Phytohabitans* are a possible source for novel and diverse polyketides and nonribosomal peptides.

## 1. Introduction

The discovery of new bioactive secondary metabolites remains one of the most important tasks for current pharmaceutical developments. Actinomycetes are well known as a potential source of diverse secondary metabolites. Numerous numbers of actinomycete strains have been isolated from soils and contributed to the discovery of useful bioactive compounds. However, it is nowadays becoming difficult to find novel compounds from soil-derived strains. That is because the majority of actinomycetal strains isolated from soil samples belong to the genus *Streptomyces* [1] and intensive exploration of *Streptomyces* strains is leading to frequent re-discovery of already reported compounds [2]. Consequently, attention has shifted from *Streptomyces* to the rare actinomycetes, especially new genera, because they are not extensively examined for the aim. New ecological niches are drawing attention as sources of new actinomycetes. The microbial flora in plant matter was reported to be different from that in soil samples and that the majority of species in such samples were rare actinomycetes [3].

Post-genomic studies for actinomycetes revealed that each actinomycete harbors in general diverse secondary metabolite-biosynthetic gene clusters (smBGCs) even if the strain had been reported to produce only few compounds [4]. This led to an approach based on analyzing smBGCs in whole genomes, called genome-mining, to search new natural products, resulting in effective isolation of new secondary metabolites [5]. Major smBGCs in actinomycetes are associated with polyketide synthase (PKS) and/or nonribosomal peptide synthase (NRPS) pathways [4,6]. Polyketide and nonribosomal peptide compounds are structurally and pharmacologically diverse and expected as a source for pharmaceutical seeds. Type-I PKSs and NRPS are large enzymes, composed of multiple catalytic domains organized into modules. Each module carries out a cycle of chain elongation, and typically contains at least three domains: a ketosynthase (KS) domain, an acyltransferase (AT) domain and an acyl carrier protein (ACP) in a PKS module; a condensation (C) domain, an adenylation (A) domain, and a thiolation (T) domain in an NRPS module. Optional domains are often present in modules to modify elongating chains chemically. The chains are synthesized from simple building blocks, such as acyl-CoA and amino-acid units by PKS and NRPS gene clusters, respectively, based on the collinearity rule of assembly-line enzymology. Therefore, chemical structures of synthesized polyketide and/or peptides can be predicted from the domain organization of these gene clusters [7].

The genus *Phytohabitans* was recently proposed as a new genus of the family *Micromonosporaceae* [8] and included four species, *Phytohabitans flavus*, *Phytohabitans rumicis*, *Phytohabitans houttuyneae*, and *Phytohabitans suffuscus*, all of which were isolated from the root tissues of plants [9], when we began this study. No genome sequences are published for this genus. Although a new meroterpenoid habiterpenol was discovered from a strain of *Phytohabitans suffuscus* [10], no polyketide and nonribosomal peptide compounds have been reported from members of the genus yet. To elucidate the capacity to synthesize polyketide and nonribosomal peptide compounds, we conducted whole genome-sequencing for type strains of these species and bioinformatically analyzed PKS and NRPS gene clusters.

## 2. Materials and Methods

### 2.1. Whole Genome Sequencing

*P. flavus* NBRC 107702^T^, *P. rumicis* NBRC 108638^T^, *P. houttuyneae* NBRC 108639^T^ and *P. suffuscus* NBRC 105367^T^ were distributed from the NBRC Couture Collection. Their genomic DNA was prepared and whole genome *de novo* sequencing was performed by Macrogen Korea, employing the SMRT strategy using PacBio RSII with SMRT cell 8Pac V3 and the DNA Polymerase Binding Kit P6, according to the procedure reported previously [11]. The reads of each strain were assembled using Canu (version 1.4). Their genome sequences were assembled into one, three, five and one scaffolds,, respectively, and have been published under the accession numbers as follows: *P. flavus* NBRC 107702^T^, AP022870; *P. rumicis* NBRC 108638^T^, BLPG01000001–BLPG0100003; *P. houttuyneae* NBRC 108639^T^, BLPF01000001–BLPF0100005; *P. suffuscus* NBRC 105367^T^, AP022871.

### 2.2. Analysis of PKS and NRPS Gene Clusters

PKS and NRPS gene clusters in the genomes were surveyed using antiSMASH and then manually analyzed in the same manner of our previous reports [12,13,14]. Subtypes of C domains were characterized with NaPDoS (http://napdos.ucsd.edu/run_analysis.html) [12]. Substrates of A domains predicted by antiSMASH were also checked using NRPSsp (http://www.nrpssp.com/execute.php) [13] and/or by the specificity-conferring codes [14]. If the same substrates are predicted between antiSMASH and NRPSsp or the selectivity-conferring codes showed >90% identity to those for the predicted amino acids, the substrates are shown in the tables. If not, the amino acids predicted by antiSMASH are shown in italics in tables. Based on the substrates, domain organization, module number, and the collinearity rule of the assembly-line enzymology [7], we predicted the chemical structures of the peptides and polyketide chains synthesized by NRPS and PKS gene clusters [15,16,17]. Norine (https://bioinfo.cristal.univ-lille.fr/norine/form2.jsp) [18] was used to search similar nonribosomal peptides.

### 2.3. LC-MS Analysis of Culture Extracts

*P. flavus* NBRC 107702^T^, *P. rumicis* NBRC 108638^T^, *P. houttuyneae* NBRC 108639^T^ and *P. suffuscus* NBRC 105367^T^ were cultured on double diluted TSA (20 g/L Bacto Tryptic Soy Agar, 7.5 g/L agar) and double diluted ISP-2 agar (2 g/L glucose, 5 g/L malt extract, 2 g/L yeast extract, 17 g/L agar) plates for 2 to 3 weeks at 28 °C. They were extracted by 3 times volume of ethanol at 4 °C overnight. One microliter of each supernatant was analyzed using an UHPLC system coupled with a mass spectrometer (LC-MS) (UltiMate 3000 UHPLC coupled with Q Exactive, Thermo Fisher Scientific K.K., Tokyo, Japan). Acquity UPLC BEH C18 1.7 μm (2.1 × 50 mm) (Nihon Waters K.K., Tokyo, Japan) was used as a reverse phase column for separation in the system. Water (solvent A) and acetonitrile (solvent B), both containing 0.1% (*v*/*v*) formic acid, were used as the mobile phase in the following linear gradient program: 5% B for 0.5 min, 5% B to 85% B in 5 min, 85% B to 100% B in 0.5min, 100% B for 2 min. The flow rate was set to 0.6 mL/min and the column oven temperature was set at 40 °C. Compounds in the eluate were detected in the electrospray ionization positive-ion mode with a spray voltage at 3.5 kV and a capillary temperature at 300 °C. Nitrogen sheath gas and auxiliary gas were set at 50 and 15 arbitrary units, respectively. A full MS scan was performed in the range of 150–2000 (*m/z*) at 70,000 resolution. Data were acquired with Xcalibur 2.0 software (Thermo Fisher Scientific K.K., Tokyo, Japan).

## 3. Results

### 3.1. Genome Analysis of Four Type Strains in the Genus Phytohabitans

Whole genomes of *P. flavus* NBRC 107702^T^, *P. rumicis* NBRC 108638^T^, *P. houttuyneae* NBRC 108639^T^ and *P. suffuscus* NBRC 105367^T^ were sequenced by PacBio. Their genome sizes were 9.6 Mb, 10.7 Mb, 11.3 Mb and 10.2 Mb, respectively. The G+C contents ranged from 70.8 to 72.0%. Each genome encoded 10 to 18 PKS and NRPS gene clusters as summarized in Table 1. Type-II PKS gene clusters were not observed in the genomes.

### 3.2. PKS and NRPS Gene Clusters Common between/among Species

**Figure 1:**
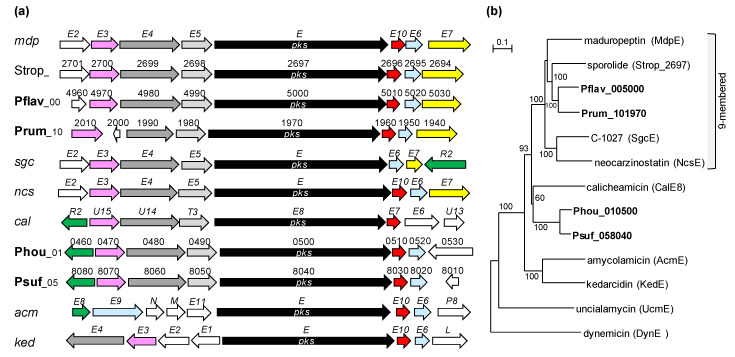
Comparison between *t1pks-1* gene clusters and reported enediyne type-I PKS gene clusters. (**a**) Gene organization. pink, DUF1702 family protein/enediyne biosynthesis protein; black, type-I PKS; red, thioesterase; light blue, flavin reductase, oxidoreductase or FAD-dependent monooxygenase; yellow, cytochrome P450; green, transcriptional regulator; white and grays, others. Homologous hypothetical genes are colored in dark gray or light gray, respectively. (**b**) Phylogenetic analysis of type-I PKSs. The phylogenetic tree was reconstructed by the neighbor-joining method using ClustalX 2.1. Numbers on the branches represent the confidence limits estimated by bootstrap analysis with 1000 replicates; values above 50% are at branching points. Accession numbers of used sequences are as follows: MdpE, AAQ17110; Strop_2697, ABP5514; SgcE, AAL06699; NcsE, AAM78012; CalE8, AAM94794; AcmE, ATV95639; KedE, AFV52145; UcmE, AMK92560; DynE, AAN79725. Kedarcidin also includes 9-membered enediyne moiety whereas the enediyne moieties of calicheamicin, uncialamycin and dynemicin are 10-membered.

Two type-I PKS (*t1pks*), two type-III PKS (*t3pks*), three NRPS (*nrps*) and one hybrid PKS/NRPS (*pks/nrps*) gene clusters were shared between/among multiple species (Table 2). *T1pks-1*, *t3pks-1* and *mrps-3* gene clusters were present in all the test strains. *T1pks-1* gene cluster encoded only one PKS gene, whose domain organization is KS/AT/KR/DH. The unusual domain organization, such as not DH-KR but KR-DH and lack of ACP, is characteristic for the iterative PKS for enediyne synthesis [19]. The organization of adjacent genes is similar to those of maduropeptin, sporolide, calicheamicin and neocarzinostatin (Figure 1a). The phylogenetic analysis of the PKSs suggested those of *t1pks-1* in *P. flavus* NBRC 107702^T^ and *P. rumicis* NBRC 108638^T^ is included in a clade of compounds with 9-membered enediyne moiety whereas those in *P. houttuyneae* NBRC 108639^T^ and *P. suffuscus* NBRC 105367^T^ are closer to that of calicheamicin, which enediyne moiety is 10-membered (Figure 1b). Thus, the products of *P. flavus* NBRC 107702^T^ and *P. rumicis* NBRC 108638^T^ will be compounds similar to these 9-member enediyne compounds. In contrast, those of *P. houttuyneae* NBRC 108639^T^ and *P. suffuscus* NBRC 105367^T^ would be different from them. *T3pks-1* gene cluster encoded one type-III PKS, which is an ortholog of *agqA* for the synthesis of alkyl-*O*-dihydrogeranyl-methoxyhydroquinones. As this cluster also encoded orthologs of non-PKS/NRPS family genes, *agqB* to *agqD* [20] (Pfav_061719 to Pfav_06721, Prum_045400 to Prum_045380, Phou_043850 to Phou_045870, Psuf_029230 to Psuf_029250), the product was deduced to be alkyl-*O*-dihydrogeranyl-methoxyhydroquinones. *Nrps-3* gene cluster resembled BGCs for siderophores, such as scabichelin and albachelin [21,22]. This cluster was predicted to synthesize a siderophore, composed of four to five amino-acid residues such as methyl-acetyl-hydroxy-ornithine (mHaOrn), methyl-ornithine (mOrn) and acetyl-hydroxy-ornithine (HaOrn) by antiSMASH analysis, although some monomers cannot be predicted. The selectivity-conferring codes [23] of A domains in the fifth modules was DAWEGGLVDK or DAWEVGLVDK, which is identical or quite similar to that of scabichelin-BGC (DAWEGGLVDK) loading hydroxy-ornithine (hOrn) [21]. *Nrps-3* gene clusters of *P. flavus* NBRC 107702^T^, *P. houttuyneae* NBRC 108639^T^ and *P. suffuscus* NBRC 105367^T^ likely share similar domain organizations (A_haorn_/MT/T–C/A/T–C/A_orn_/MT/T–C/A_haorn_/T–C/A/T/E), whereas that of *P. rumicis* NBRC 108638^T^ lacked C/A/T of the last module. Therefore, the products will be different between the three strains and *P. rumicis* NBRC 108638^T^, which are predicted as mHaOrn-x-mOrn-HaOrn-hOrn and mHaOrn-x-mOrn-HaOrn, respectively. *Pks/nrps-3* gene cluster was present in three strains except for *P. rumicis*. This cluster harbors ten KR domains and eleven DH-KR domain pairs, catalyzing hydroxy groups and C=C double bond formations [7], respectively, were present as optional domains. Therefore, the product will be a large polyene compound, although it is unpredictable how the A domain in the last ORF is involved in the synthesis. It may suggest a novel function of A domain. *T1pks-4* and *nrps-4*, *t3pks-4* and *nrps-11*, and *nrps-2* gene clusters were distributed between *P. rumicis* and *P. houttuyneae*, between *P. houttuyneae* and *P. suffuscus*, and between *P. flavus* and *P. rumicis*, respectively. *T1pks-4* gene cluster encoded one PKS with single module, whose polyketide backbones were not predicted by their domain organization. *Nrps-4* genes did not show high sequence similarities to published genes whose products are identified. However, based on the module numbers, the products were deduced to be a small molecule. *T3pks-4* gene cluster encoded a type-III PKSs and combined with a terpenoid gene cluster. The hybrid cluster resembled diazepinomicin BGC, which suggests being responsible for the synthesis of the compound. *Nrps-11* gene cluster did not show high sequence similarities to published genes whose products are identified. However, based on the domain organization, the product was deduced to be a nonapeptide, in which some amino acid residues may be modified by multiple C domains that were predicted to be involved in modification of the incorporated amino acid residues (C_M_). *Nrps-2* gene cluster encoded seven NRPSs. According to the domain organization, which assembly line is T–C/A/T–C/A_asn_/T–C/A_ser_/T–C/T–C/A_asn_/T–C/A_gly_/T, the product is predicted to be a heptapeptide including two Asn, one Ser and one Gly residues. Nonribosomal peptides similar to those of *nrps-2*, *-4* and *-11* were not found in our database search using Norine.

### 3.3. PKS and NRPS Gene Clusters Specific to Each Strain

#### 3.3.1. *P. flavus* NBRC 107702^T^

Five gene clusters were specific to *P. flavus* NBRC 107702^T^ (Table 3). *T1pks-2* gene cluster encoded AT-less PKSs. The domain organization of Pfav_13200 to Pfav_13340 was similar to that of PKS for anthracimycin synthesis [23]. However, this cluster encodes additional PKSs (Pfav_012890 to Pfav_012980), which are not present in the anthracimycin biosynthetic gene cluster (BGC). Thus, the product was predicted to be a larger polyketide than anthracimycin, which includes an anthracimycin-like moiety as a part. *T1pks/t3pks* was a hybrid gene cluster encoding 21 type-I PKSs and one type-III PKS. The type-I PKS harbors five KR domains and nine DH-KR domains. Hence, the product will be a large polyene compound whose starter unit is a chalcone-like moiety derived from type-III PKS. *Nrps-1* gene cluster harbored less than two modules. Hence, the product would be simple. *Pks/nrps-1* gene cluster encoded two hybrid PKS/NRPS proteins. They are predicted to form only two modules, which load AHBA and methylmalonyl-CoA, respectively. The molecule synthesized by this cluster would be small and simple. *Pks/nrps-2* gene cluster encoded six NRPSs and one PKS. They included one loading module, one PKS module and five NRPS modules and it is deduced to synthesize a peptide containing one Asp and two Ser molecules and a polyketide unit. Hybrid polyketide/nonribosomal peptide compounds resembling that of *pks/nrps-2* were not found in our database search.

#### 3.3.2. *P. rumicis* NBRC 108638^T^

One *t1pks*, two *t3pks*, three *nrps* and two *pks*/*nrps* gene clusters were specific to *P. rumicis* NBRC 108638^T^ (Table 4). *T1pks-3* gene cluster resembled pyrrolomycin BGC and their domain organizations were the same. Therefore, it will be responsible for the synthesis of pyrrolomycin. The products of *t3pks-2* and *-3* gene clusters were not able to be predicted by this bioinformatic analysis. However, as *t3pks-2* gene cluster also encoded terpenoid-biosynthetic genes, we predicted the product to be a terpenoid with a polyketide moiety derived from type-III PKS. The products of *nrps-5*, *-6* and *-7* gene clusters were predicted to be tetrapeptides as shown in Table 4. Similarly, the products of *pks/nrps-4* and *-5* were deduced to be tetra- and penta-peptides, respectively, with a polyketide moiety. Nonribosomal compounds like those of *nrps-5, nrps-6, nrps-7, pks/nrps-4* and *pks/nrps-5* were not found in our database search.

#### 3.3.3. *P. houttuyneae* NBRC 108639^T^

Two *t1pks*, one *t3pks*, five *nrps* and three *pks*/*nrps* gene clusters were specific to *P. houttuyneae* NBRC 108639^T^ (Table 5). *T1pks-5* encoded one PKS with single module, whose polyketide backbones were not predicted by their domain organization. *T1pks-6* gene cluster showed similarity to deschlorothricin-BGC. However, as their domain organizations were different each other, the product of *t1pks-6* will not be deschlorothricin but a deschlorothricin-like compound. Since *t3pks-4* gene cluster did not show high amino acid sequence similarities to product-identified gene clusters, its product was not able to be speculated. *Nrps-8, -9, -10* and *-12* gene clusters also did not show high similarities to gene clusters whose products are identified. However, according to the domain organizations and/or substrates of A domains, their products were deduced to be di-, nona-, penta-, and penta-peptides, respectively, as shown in Table 5. *Pks/nrps-7* gene cluster encoded ten NRPSs harboring multiple domains, forming 24 NRPS modules, and one type-III PKS. Hence, it will synthesize a large peptide composed of 24 amino-acid residues with a polyketide moiety derived from type-III PKS. In contrast, *pks/nrps-6* and *-8* gene clusters encoded less NRPSs and their products were predicted to be tri-, and tetra-peptides with a moiety derived from each small PKS, respectively. Nonribosomal peptide- and/or hybrid polyketide/nonribosomal peptide-compounds shown as deduced product in Table 5 were not found in our database search.

#### 3.3.4. *P. suffuscus* NBRC 105367^T^

One *t1pks*, four *nrps* and four *pks*/*nrps* gene clusters were specific to *P. suffuscus* NBRC 105367^T^ (Table 6). *T1pks-7* gene cluster encoded 16 PKS proteins, whose modules were twelve. Since there are six DH-KR pairs, yielding C=C double bonds, this product will be a polyene polyketide. *Nrps-14* was assigned to be a BGC for pentapeptides as shown in Table 6. As *nrps-14* gene cluster was similar to BGC for cephamycin, we considered it to be a cephamycin BGC. *Nrps-15* gene cluster encoded four NRPSs, two of which included a terminal TE domain, respectively. Although it is unclear which TE of the two is functional, we predicted the product to be DHB-Ser based on ORFs of Psuf_002170, Psuf_002160 and Psuf_002150. Such a part is often observed in siderophores. By catalyzing iteratively, this cluster may synthesize a siderophore like enterobactin, which is composed of three pairs of DHB-Ser. *Pks/nrps-9* gene cluster encoded one iterative PKS for enediyne and one type-III PKS in addition to 13 NRPSs for a total number of module of six. The product would be a hexapeptide including Ser, Cys, Pro as the amino-acid residues, a polyketide component derived from type-III PKS, and an enediyne moiety. Although the product of *pks*/*nrps-10* gene cluster was unclear, it will be a polyketide with a thiazoline residue formed by cyclization of Cys. *Pks/nrps-11* gene cluster was considered to be a chlorizidine BGC according to the similarity between their gene organizations. *Pks/nrps-12* gene cluster encoded 22 proteins, whose PKS modules were 13, and one NRPS. In the PKS domain organization, four KR domains and four DH-KR domain pairs were present, suggesting the product to be a polyene compound with a moiety derived from Leu. Deduced products of *nrps-13, pks/nrps-9, pks/nrps-10* and *pks/nrps-12* were not reported in our database search.

### 3.4. Genomic Positions of the Gene Clusters

**Figure 2:**
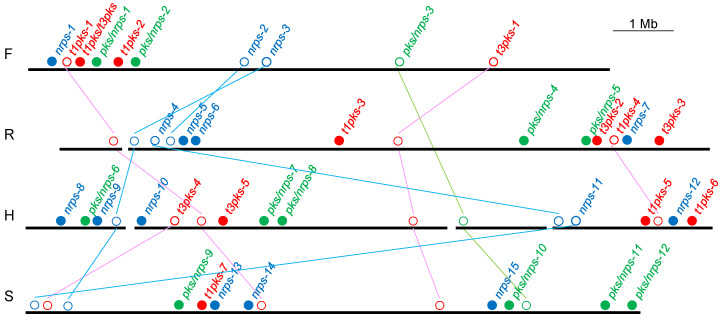
Positions of PKS and NRPS gene clusters in chromosomes of (**F**) *P. flavus* NBRC 107702^T^, (**R**) *P. rumicis* NBRC 108638^T^, (**H**) *P. houttuyneae* NBRC 108639^T^ and (**S**) *P. suffuscus* NBRC 105367^T^. Chromosome or scaffold sequences are indicated by black and bold horizontal lines. Red, PKS gene cluster; blue, NRPS gene cluster; green, hybrid PKS/NRPS gene cluster. The cluster numbers are the same as those in Table 2, Table 3, Table 4, Table 5 and Table 6. Gene clusters specific to each strain are shown by filled circles. Orthologous gene clusters are shown by open circles and connected between strains by lines. Alignments and direction of scaffold sequences in R and H are putative because their whole genome sequences are not complete.

Genomic positions of the PKS and NRPS gene clusters were diagrammatically shown in Figure 2. Orthologous clusters present between/among the strains are connected by line in the figure. All the strains harbored *t1pks-1, t3pks-1* and *nrps-3* gene clusters. *Pks/nrps-3* were present in three strains except for *P. rumicis* NBRC 108638^T^. *Nrps-4* and *t1pks-3* were distributed between *P. rumicis* NBRC 108638^T^ and *P. houttuyneae* NBRC 108639^T^, whereas *t3pks-4* and *nrps-11* were between *P. houttuyneae* NBRC 108639^T^ and *P. suffuscus* NBRC 105367^T^. The remaining 31 gene clusters were not shared between different species: five, eight, ten and eight were specific to *P. flavus* NBRC 107702^T^, *P. rumicis* NBRC 108638^T^, *P. houttuyneae* NBRC 108639^T^ and *P. suffuscus* NBRC 105367^T^, respectively, as shown by closed circles in the figure.

### 3.5. Production of Unknown Compounds

The four strains were cultured on two kinds of agar medium. The growths of *P. flavus* NBRC 107702^T^ and *P. rumicis* NBRC 108638^T^ were poor compared with the other two strains. The culture extracts were analyzed by LC-MS. Ion peaks corresponding to [M+H]^+^ of known compounds, such as alkyl-*O*-dihydrogeranyl-methoxyhydroquinones (exact mass; 486.41, 500.42, 556.49, 570.50), diazepinomicin (462.25), pyrrolomycins (353.91, 322.91, 305.94 etc.), cephamycins (579.15, 446.11, 659.11), enterobactin (669.14) and chlorizidines (441.94, 415.97), were not observed. In contrast, the other ion peaks were observed, among which we here picked up ones listed in Table 7. Ion peaks of *m/z* 656.31 eluted at 2.8 min (**3**) were observed in the culture extracts of the three strains except for *P. rumicis* NBRC 108638^T^ whereas ion peaks of **1** and **2** were observed specifically in that of *P. rumicis* NBRC 108638^T^. Ion peaks (**4**) and (**5**) were specific for *P. suffuscus* NBRC 105367^T^ and *P. houttuyneae* NBRC 108639^T^, respectively. We searched reported compounds with these accurate mass values in the database of Dictionary of Natural Products and consequently there are not significant hits, suggesting that these compounds are likely novel.

## 4. Discussion

In conventional screenings for novel secondary metabolites, re-isolation of known compounds has been problematic. This caused a change in the strategies used for natural product discovery by shifting to new sources as producing microorganisms. Prediction of products based on smBGCs, such as PKS and NRPS gene clusters, is a powerful approach to reduce the frequency to isolate known compound although further investigations need to be carried out.

Here, we sequenced whole genomes in four type strains of the genus *Phytohabitans*, which have not been studied by genome sequence-based strategies, by PacBio, analyzed their PKS and NRPS gene clusters and bioinformatically predicted the chemical structures of the products derived from these gene clusters. Fifty-six gene clusters were identified from the four strains, which are involved in the biosynthesis of 40 different types of polyketide and/or nonribosomal peptide compounds. Although analysis focusing on the domain organizations and bioinformatical substrate prediction is not sufficient to conclude the products are the same because of possible variants derived from low substrate selectivity of A and AT domains, few gene clusters were shared between/among different species. Each strain harbored five to eleven specific gene clusters. Most of the gene clusters are not for known compounds and their predicted chemical structures are novel. Among the 40 biosynthetic gene clusters, only six were identified to produce known products. These known compounds were not produced in our culture conditions. These BGCs may be cryptic in the condition and/or their productivity may too low to detect them in samples derived from small scale cultures. To express these BGGs and/or produce more, further investigations are necessary. The duplication of the putative metabolites within the genus were only nine as shown in Table 2, suggesting many are specific in each species. Therefore, members of the genus *Phytohabitans* are considered as an attractive source for novel and diverse secondary metabolites. To confirm it, we analyzed the culture extracts by LC-MS as a preliminary study. As expected, ion peaks corresponding to some putative novel compounds were observed. We are guessing that the products of ion peaks **1** to **3** are siderophores derived from *nrps3*. *Nrps-3* gene cluster is distributed to the four strains but that in *P. rumicis* NBRC 108638^T^ lacks the fifth module and its product will be smaller than the others. *Nrps-3* resembles that of scabichelin, whose exact mass is 647.36. Although the second amino acid residue of the product by *nrps-3* was unpredictable in this study, the exact mass will be close to that of scabichelin because they are similar siderophores. Thus, the compound of *m/z* 656.31 is plausible as the product. Furthermore, *P. rumicis* NBRC 108638^T^, whose *nrps-3* lacks the fifth module, did not produce the product of *m/z* 656.31, but produced smaller ones, which can be account for by the absence of the fifth module. Unfortunately, it is not possible to determine chemical structures of final products because PKS and NRPS assembly lines determine chemical structures of the backbones [7], but do not those of final products since the backbones are usually modified by other enzymes to yield the final products. It is unclear at present which gene clusters in *P. suffuscus* NBRC 105367^T^ and *P. houttuyneae* NBRC 108639^T^ synthesize the two putative novel compounds (**4**, **5**). Except for siderophores, remarkable ion peaks with high intensities were not observed from *P. flavus* NBRC 107702^T^ and *P. rumicis* NBRC 108638^T^. This may be due to their poor growth in our culture conditions.

Compared with general type-I PKSs and NRPSs, some of those in the genus *Phytohabitans* were observed to split on many proteins. It is still unclear if it is artifact from sequencing and/or assembly technologies. However, obvious ORFs that are likely involved in biosynthetic pathway, such as accessory enzymes, were not observed between such the split PKS and NRPS genes. To confirm whether modular enzyme genes are often split in the genus *Phytohabitans* or whether it is due to technological artifact(s), more reliable method(s) should be employed.

During this study, a novel species *Phytohabitans kaempferiae* was reported, which is an endophytic actinomycete isolated from the leaf of *Kaempferia larsenii* [24]. Although the whole genome has yet to be sequenced, the analysis will also reveal further potential of the genus because different species, in general, harbor specific PKS and NRPS pathways, as shown in this and our previous studies on actinomycetes [15,16,17].

## Figures and Tables

**Table 1:** Genomic features of strains used in this study.

Strains	Genome Size (Mb)	G+C Content	Number of Gene Clusters					
			PKS			NRPS	Hybrid PKS/NRPS	Total
			I	III	I/III			
*P. flavus* NBRC 107702^T^	9.61	70.8%	2	1	1	3	3	10
*P. rumicis* NBRC 108638^T^	10.71	71.1%	3	3	–	6	2	14
*P. houttuyneae* NBRC 108639^T^	11.34	71.6%	4	3	–	7	4	18
*P. suffuscus* NBRC 105367^T^	10.15	72.0%	2	2	–	5	5	14

**Table 2:** Orthologous PKS and NRPS gene clusters between/among *Phytohabitans* species.

Gene Cluster	ORF (Locus Tag)				Domain Organization *	Predicted Product
	Pflav_	Prum_	Phou_	Psuf_		
*t1pks-1*	005000	101970	010500	058040	KS/AT_m_/KR/DH	enediyne-compounds
*t1pks-4*	n/a	07110	055560	n/a	KS/AT_p_/DH/ER/KR/ACP	unknown
*t3pks-1*	067180	045410	043880	029220	KS (type-III PKS)	alkyl-*O*-dihydrogeranyl-methoxyhydroquinones
*t3pks-4*	n/a	n/a	005750	091470	KS (type-III PKS)	diazepinomicin
*nrps-2*	032950	078990	n/a	n/a	T	s-x-Asn-Ser-x-Asn-Gly
	032940	079000			^L^C_L_	
	032910	079030			A/T–^L^C_L_	
	032880	079150			A_asn/_T	
	032770	079170			^L^C_L_/A_ser_/T–C_M_/A	
	032760	079180			T–C_M_/A_asn_/T/–C_M_	
	032750	079190			A_gly_/T	
*nrps-3*	036450	084390	072920	088300	A_haorn_/MT/T–^D^C_L_/A/T–	siderophores such as mHaOrn-x-mOrn-HaOrn(-hOrn)
	to	to		to	C_D_/A_orn_/MT/T–^L^C_L_/A_haorn_	
	036480	084420		088260	/T–^D^C_L_/A/T/E **	
*nrps-4*	n/a	080570	069630	n/a	FkbH/T	s-y-Asx
		080550	069620		^L^C_L_ or ^L^C_L_/T	
		080540	069610		T or ^L^C_L_	
		080520	069600		^L^C_L_/A_asp_/T–TE or A_asn_/T	
					–TE	
*nrps-11*	n/a	n/a	067470	092920	T	s-x-x-Thr-x-x-*Orn*-x-*Ala*-Asn
			067480	092910	^L^C_L_	
			067520	092870	A/T–^L^C_L_/A/T	
				–092860		
			067530	092850	^L^C_L_/A_thr_/T–C_M_/A/T–C_M_/A	
					/T–C_M_/A*_orn_*/T–C_M_	
			067540	092840	T–C_M_/A*_ala_*/T–C_M_/A_asn_/T	
				–092820		
*pks/nrps-3*	57090	n/a	95390	16680	KS/AT_m_/DH/KR/ACP	Large polyene
	to		to	to	–KS/AT_m_/KR/ACP	
	57060		95400	16670	–KS AT_p_/KR/ACP	
					–KS AT_m_/KR/ACP	
					–KS/AT_m_/KR/ACP	
					–KS/AT_m_/KR/ACP	
	57050		95410	16660	KS/AT_m_/DH/ER/KR/ACP	
	to		to	to	–KS/AT_m_/KR/ACP	
	57020		95440	16600	–KS/AT_m_/DH/KR/ACP	
					–KS/AT_m_/DH/KR/ACP	
	57010		95450	16590	KS/AT_m_/DH/ER/KR/ACP	
	–57000			–16580		
	56970		95480	16550	A	
	56960		95490	16540	AT	
	56950		95500	16530	ER	
	56890		95550	16480	ACP	
	to		to	to	–KS/AT_m_/DH/KR/ACP	
	56870		95570	16470	–KS/AT_m_/DH/ER/KR/ACP	
	56860		95580	16460	KS/AT_m_/DH/ER/KR/ACP	
	–56840		–95600	–16450	–KS/AT_m_/DH/KR/ACP	
	56830		95610	16430	KS/AT_m_/KR/ACP	
	to		to	to	–KS/AT_m_/KR/ACP	
	56820		95640	16420	–KS/AT/KR/ACP	
					–KS/AT_p_/KR/ACP	
	56810		95650	16410	KS/AT_m_/DH/KR/ACP	
	to			to	–KS/AT_m_/DH/KR/ACP	
	56790			16390	–KS/AT_m_/DH/KR/ACP	
					–KS/AT_m_/DH/KR/ACP	
					–KS/AT_m_/DH/KR/ACP	
	56780		95660	16380	KS/AT_m_/DH/KR/ACP	
				–16350		
	56630		95800	16270	ACP	
	56620		95810	16260	KS	
	56600		95830	16240	A–TE	

* Typical domain organizations are shown as slight differences were observed between/among strains. ** Domain organization of *nrps-3* gene cluster in *P. rumicis* NBRC 108638^T^ was A/MT/T–C_M_/A/T–C_M_/A/MT/T–C_L_/A/T/E. Slash is inserted between domains, whereas hyphen is between modules. Amino acid residues in italics were predicted only by antiSMASH. Abbreviations: A, adenylation; Aad, 2-aminoadipic acid; ACP, acyl carrier protein: AHBA, aminohydroxybenzoate; Asx, Asp or Asn; AT, acyltransferase; AT_e_, AT for ethylmalonyl-CoA; AT_m_, AT for malonyl-CoA; AT_p_, AT for methylmalonyl-CoA; C, condensation; C_Cy_, heterocyclization (Cyc) domain that catalyze both peptide bond formation and subsequent cyclization of cysteine, serine or threonine residues.; ^D^C_L_, C domain that link an L-amino acid to a growing peptide ending with a D-amino acid; C_Du_, dual E/C domain that catalyze both epimerization and condensation; ^L^C_L_, C domain that catalyze a peptide bond between two L-amino acids; C_M_, C domain that appears to be involved in the modification of the incorporated amino acid, for example the dehydration of serine to dehydroalanine; C_S_, starter C domain (first dominated and classified as a separate subtype here) which acylates the first amino acid with a β-hydroxy-carboxylic acid (typically a β-hydroxyl fatty acid); CoL, CoA-ligase; DH, dehydratase; Dha, dehydroalanine; DHB, hydroxybenzoate; dVal, D-Val; dx, D-amino acid; E, epimerase; ER, enoylreductase; HaOrn, acetyl-hydroxy-Orn; hOrn, hydroxy-Orn; KS, ketosynthase; KS_1_, KS domain present in the first module of assembly lines; KR, ketoreductase; mHaOrn, methyl-HaOrn; mOrn, methyl-Orn; MT, methyltransferase; mx, methyl-amino acid; n/a, not present; Orn, ornithine; pk, polyketide moiety; s, starter molecule; T, thiolation (peptidyl carrier protein); TE, thioesterase; TD, termination domain; x, unidentified amino acid residue; y, unknown residue due to the lack of A domain. Inferior 3-letter-abbriviated amino acids just after A domains are substrates of the A domain.

**Table 3:** PKS and NRPS gene clusters specific to *P. flavus* NBRC 107702^T^.

Gene Cluster	ORF (Pflav_)	Size (aa)	Domain Organization	Deduced Product
*t1pks-2*	012890	2993	KS/DH/KR/ACP–KS/KR/ACP–KS	large polyketide with anthracimycin-like moiety
	012900	3001	DH/ACP–KS/DH	
	012920	2707	KR/ACP–KS/ACP–KS/DH/KR/ACP–KS	
	012930	832	ACP–KS	
	012940	820	KR/ACP	
	012950	1099	KS/DH/KR	
	012960	1301	MT/ACP–KS/DH/ACP	
	012970	535	KS	
	012980	117	ACP	
	013200	893	AT_m_/AT_m_	
	013220	3254	KS/KR/ACP–KS/DH/KR/ACP–KS	
	013230	1768	DH/KR/MT/ACP	
	013240	985	ER–KS/DH	
	013260	1140	ACP–KS/DH	
	013270	1303	KR/ACP–KS/ACP	
	013280	892	KS/DH	
	013290	909	KR/MT	
	013300	320	KS	
	013310	1302	DH/KR/ACP	
	013320	833	KS/ACP	
	013220	554	KS	
	013340	756	ACP/TE–MT	
*t1pks/t3pks*	008030 *	369	KS (type-III PKS)	large polyene with a starter derived from type-III PKS
	008050	2178	CoL/KR/ACP–KS/AT_p_/DH	
	008060	3857	KR/ACP–KS/AT_p_/DH/KR/ACP	
			–KS/AT_m_/KR/ACP	
	008070	3045	KS/AT_p_/DH/ER/KR/ACP–KS/AT_m_	
	008080	4076	KR/ACP–KS/AT_m_/DH/KR/ACP	
			–KS/AT_m_/DH/KR/ACP	
	008090	5897	KS/AT_p_/DH/KR/ACP–KS/AT_m_/KR/ACP	
			–KS/AT_p_/DH/KR/ACP–KS/AT_m_/ACP	
	008100	377	KS	
	008110	1236	AT_p_/DH/KR/ACP	
	008130	250	KS	
	008140	865	AT_m_/DH	
	008150	419	ACP–KS	
	008190	3436	KR/ACP–KS/AT_m_/DH/KR/ACP	
			–KS/AT_m_/DH	
	008200	1003	ER/KR/ACP	
	008210	425	KS	
	008220	490	AT_p_	
	008240	1407	DH/KR/ACP–KS	
	008250	2346	AT_m_/KR/ACP–KS/AT_m_/KR/ACP	
	008260	319	KS	
	008270	2499	AT_m_/DH/KR/ACP–KS/AT_m_/DH	
	008280	1703	ER/KR/ACP–KS/AT_m_	
	008290	976	ER/KR	
	008300	356	ACP/TE	
*nrps-1*	004670 *	93	T	s-x
	004680	1055	C_L_/A/T	
	004700	452	C_L_	
*pks/nrps-1*	010020	1437	CoL_AHBA_/ACP–KS/AT_p_	AHBA-pk
	010030	551	ACP–^L^C_L_	
*pks/nrps-2*	015420	1122	T–^L^C_L_/A/T	s-x-pk-Asp-y-Dha-Dha
	015410	1831	KS/AT_m_/KR/DH/ACP	
	015390	284	^L^C_L_	
	015380	1445	A_asp_/T–^L^C_L_	
	015370	725	T–C_M_	
	015360	780	A_ser_/T	
	015350	1329	C_M_/A_ser_/T–TE	

* Encoded in the complementary strand. Abbreviations are the same as those of Table 2.

**Table 4:** PKS and NRPS gene clusters specific in *P. rumicis* NBRC 108638^T^.

Gene Cluster	ORF (Prum_)	Size (aa)	Domain Organization	Deduced Product
*t1pks-3*	052570	376	KS	pyrrolomycin
	052590	423	KS	
	052640	1481	AT_m_/ACP–KS/ACP	
	052650	2081	KS/AT_m_/DH/KR/ACP–TD	
*t3pks-2*	009490	356	KS (type-III PKS)	terpenoid with pk moiety
*t3pks-3*	005640	359	KS (type-III PKS)	unknown
*nrps-5*	073950 *	497	A	x-y-Cys-x
	074040 *	89	T	
	074060	556	^L^C_L_/T	
	074070	563	^L^C_L_	
	074090	1538	T–C_M_/A/T–TE	
	074100	544	A_cys_	
*nrps-6*	073810 *	2114	^L^C_L_/A_phe_/T–^L^C_L_/A/T	Phe-x-Ser-x
	073870	2381	^L^C_L_/A_ser_/T–C_M_/A/T–TE	
*nrps-7*	005920	514	A	x-Gly-Gly-x
	006040 *	865	T–TE	
	006050 *	971	^L^C_L_/A	
	006060 *	1054	^L^C_L_/A_gly_/T	
	006070 *	450	^L^C_L_	
	006090	541	^L^C_L_	
	006100	464	A_gly_/T	
	006110	85	T	
*pks/nrps-4*	021280	1995	C_S_/A/T–^L^C_L_/A_phe_	x-Phe-x-Phe-pk
	021270	2780	T–^L^C_L_/A/T/E–^D^C_L_/A_phe_/T	
	021190	1102	KS/AT	
	021180	410	KR/ACP	
	021250	443	ER	
*pks/nrps-5*	011340	1837	KS/AT_m_/KR/DH/ACP	pk-*Ser*-x-Ser-Gly-x
	011290 *	2046	A_gly_/T–^L^C_L_/A	
	011280	887	^L^C_L_/A*_ser_*	
	011270	317	T–^L^C_L_	
	011260	831	A/T	
	011250	1718	^L^C_L_/A_ser_/T–C_M_	

* Encoded in the complementary strand. Abbreviations are the same as those of Table 2.

**Table 5:** PKS and NRPS gene clusters specific to *P. houttuyneae* NBRC 108639^T^.

Gene Cluster	ORF (Phou_)	Size (aa)	Domain Organization	Deduced Product
*t1pks-5*	056050	2029	KS/AT_m_/DH/ER/KR/ACP	unknown
*t1pks-6*	049460 *	1704	KS_1_/AT/DH/KR/ACP	deschlorothricin-like polyketide
	049580	3493	KS/AT_m_/ACP	
			–KS/AT_m_/DH/KR/ACP	
			–KS/AT_e_	
	049590	1347	DH/ER/KR/ACP	
	049600	3825	KS/AT_p_/DH/KR/ACP	
			–KS/AT_m_/DH/ER/KR/ACP	
	049630	3144	DH/KR/ACP	
			–KS/AT_p_/DH/ER/KR/ACP	
	049640	1512	KS/AT_p_/KR/ACP	
	049700 *	1537	KS/AT_m_/ACP	
	049740 *	820	DH/KR	
	049750 *	841	KS/AT_m_	
	049760 *	2148	KS/AT_p_/DH/ER/KR/ACP	
	049770 *	2263	KR/ACP–KS/AT_p_/KR/ACP	
	049780*	2211	KS/AT_m_/KR/ACP–KS	
*t3pks-4*	014220	245	KS (type-III PKS)	unknown
*nrps-8*	083430	1179	C_S_/A/T	x-x
	083460	528	A	
*nrps-9*	077860	1332	A/T–^L^C_L_/A	x-x-dVal-dVal-x-dx-Thr-Val-x
	077870	4920	T/E–^D^C_L_/A_val_/T/E–^D^C_L_/A_val_/T/E–^D^C_L_/A/T	
	077880	2628	^L^C_L_/A/T/E–^D^C_L_/A_thr_/T–^L^C_L_	
	077890	3704	A/T/E–^D^C_L_/A_val_/T–^L^C_L_/A/T/E	
*nrps-10*	004780	1019	^L^C_L_/A_gly_/T	pentapeptide containing Gly and Cys
	004790	569	A/T	
	004800	167	T	
	004810*	2225	A_cys_/T–TE–^L^C_L_/A/T	
	004820*	628	^L^C_L_	
	004830*	463	^L^C_L_	
	004840	413	A	
*nrps-12*	052350 *	590	A_val_/T	Val-x-y-x-x
	052430	606	A/T	
	052440	1105	^L^C_L_/T–^L^C_L_	
	052470	611	A/T	
	052540	477	^L^C_L_	
	052560	682	A/T–TE	
*pks/nrps-6*	078860 *	184	ER	*Ala*-Leu-x-pk
	078970	407	A*_ala_*	
	078980	1282	T–^L^C_L_/A_leu_/T	
	078990	1277	^L^C_L_/A	
	079000	236	T	
	079010	759	KS/AT_m_	
	079030	94	ACP	
	079050	359	FkbH	
	079160	284	KR	
*pks/nrps-7*	020430	3673	^L^C_L_/A/T–^L^C_L_/A_asp_/T–^L^C_L_/A/T–^L^C_L_	x-Phe-Asp-Asp-x-Asp-x-Gly-*Leu*-Tyr-Thr-x-Asp-Gly-Asp-x-x-x-x-Thr-Tyr-Asp-Tyr-Asp with pk
	020420	1353	A_gly_/T–^L^C_L_/A*_leu_*	
	020410	3833	T–^L^C_L_/A_tyr_/T–^L^C_L_/A_thr_/T–C_Du_/A/T–^L^C_L_	
	020400	2741	A_asp_/T–^L^C_L_/A_gly_/T–^L^C_L_/A_asp_/T	
	020370	4620	C_Du_/A/T–^L^C_L_/A/T–C_Du_/A/T–^L^C_L_	
	020360	1578	A_thr_/T–^L^C_L_/A_tyr_	
	020350	459	T–^L^C_L_	
	020340	3163	A_asp_/T–^L^C_L_/A_tyr_/T–^L^C_L_/A_asp_/T–TE	
	020260	2056	C_S_/A_phe_/T–^L^C_L_/A_asp_/T	
	020250	1041	^L^C_L_/A_asp_/T	
	020190	375	KS (type-III PKS)	
	020170*	492	A	
	020130*	106	T	
	020120	104	T	
*pks/nrps-8*	022380	471	^L^C_L_	x-Gly-pk-Gly-x
	022350	1042	^L^C_L_/A_gly_/T	
	022330	661	^L^C_L_	
	022320	398	T	
	022310	872	T–KS/AT_m_	
	022300	1041	DH/KR/ACP	
	022280 *	1004	^L^C_L_/A_gly_/T	
	022270	87	T	
	022260 *	506	A	
	022210	296	A	
	022190	507	T–^L^C_L_	
	022170 *	352	T–TE	

* Encoded in the complementary strand. Abbreviations are the same as those of Table 2.

**Table 6:** PKS and NRPS gene clusters specific in *P. suffuscus* NBRC 105367^T^.

Gene Cluster	ORF (Psuf_)	Size (aa)	Domain Organization	Deduced Product
*t1pks-7*	066740	406	KS_1_	polyene derived from C_24_ polyketide chain
	066760	1871	AT_m_/ACP–KS/AT_p_/DH/KR	
	066770	2858	ACP–KS/AT_p_/DH/KR/ACP–KS/AT_m_/KR	
	066780	1414	ACP–KS/AT_p_/DH	
	066790	380	KR/ACP	
	066800	2229	KS/AT_p_/DH/KR/ACP–KS	
	066810	1470	AT_p_/DH/ER/KR/ACP	
	066820	235	KS	
	066830	433	AT_m_	
	066840	986	DH/KR/ACP	
	066850	1844	KS/AT_p_/DH/KR/ACP	
	066860	764	KS/AT_p_	
	066870	230	KR	
	066880	282	ACP	
	066890	702	KS	
	066900	655	KR	
	066910	2409	ACP–KS/AT_e_/DH/ER/KR/ACP–TE	
*nrps-13*	065520 *	922	^L^C_L_/A	s-x-*Leu*-x-y-Ser
	065480 *	675	A*_leu_*/T	
	065440	1103	T–^L^C_L_/T	
	065390 *	466	A/T	
	065370	753	^D^C_L_/A_ser_	
	065360	291	T	
	065270	792	A*_leu_*/T	
	065190	2256	CoL/T–^L^C_L_/A/T–^L^C_L_	
*nrps-14*	059700	3735	A_aad_/T–^D^C_L_/A_cys_/T–^L^C_L_/A_val_/T/E–TE	cephamycin
*nrps-15*	0021240 *	852	A/T–TE	DHB-Ser
	0021170	556	A_dhb_	
	0021160	299	T	
	0021150	1293	C_S_/A_ser_/T–TE	
*pks/nrps-9*	070000	1920	KS/AT_m_/KR/DH	hexapeptide including Ser, Cys, Pro, pk and enediyne-moiety
	070250 *	382	KS (type-III PKS)	
	070260 *	463	^L^C_L_	
	070270 *	588	A/T	
	070280	533	T–^L^C_L_	
	070350	1174	A_ser_/T–^L^C_L_	
	070360	361	A/T	
	070410	872	A_cys_/T	
	070420	534	T–^L^C_L_	
	070430	439	^L^C_L_	
	070440	97	T	
	070450	527	A	
	070460	451	^L^C_L_	
	070490	532	A_pro_	
	070520	89	T	
*pks/nrps-10*	018900 *	747	ER/KR/ACP	polyketide including a thiazoline residue
	018190 *	1328	KS/AT_p_/DH	
	018880 *	1251	AT_p_/KR/ACP	
	018870 *	1643	KS/AT/ACP–KS	
	018860	1779	KS/ACP–C_Cy_/A_cys_/T	
	018830	384	AT_m_	
	018820	606	ACP–KS	
	018810	1588	AT_p_/DH/ER/KR	
	018800	353	KS	
	018790	791	AT_p_	
	018780	362	KR/ACP	
	018750 *	87	ACP	
	018720	492	A	
*pks/nrps-11*	004960	150	AT_m_	chlorizidine
	005010 *	501	A	
	005100	1489	KS/AT/ACP–KS	
	005110	1223	AT/DH/KR	
	005120	123	ACP	
	005130	294	KS	
	005140	380	KS	
	005150	795	AT_p_/ACP–TD	
	005190	85	ACP	
	005210	333	KS	
	005190	1358	A_gln_/T	
*pks/nrps-12*	000760	602	A_leu_/T	likely polyene with Leu
	000690	1757	KS/AT/DH/KR/ACP	
	000570	399	KS	
	000560	1292	AT_p_/DH/KR/ACP	
	000540	335	AT_p_	
	000530	967	DH/ER/KR	
	000520	206	ACP	
	000510	1267	KS/AT_p_	
	000500	237	ACP	
	000460 *	90	ACP	
	000400 *	911	KS/ACP	
	000370 *	1379	AT_p_/DH/KR/ACP	
	000360 *	409	KS	
	000350 *	2152	KS/AT_p_/DH/ER/KR/ACP	
	000330 *	2862	KS/AT_m_/KR–KS/AT_m_/KR/ACP	
	000320 *	1727	KS/AT_m_/KR/ACP	
	000250	2982	KS_1_/AT/ACP–KS/AT_p_/KR/ACP–KS	
	000240	617	AT_p_/DH	
	000230	553	KR	
	000220	2051	KS/AT_m_/DH/ER/KR/ACP	
	000180 *	448	ER	
	000160 *	89	ACP	

* Encoded in the complementary strand. Abbreviations are the same as those of Table 2.

**Table 7:** Representative ion peaks in the LC-MS analysis.

Retention Time	Observed Ion Peak (*m/z*)			
	*P. flavus* NBRC 107702^T^	*P. rumicis* NBRC 108638^T^	*P. houttuyneae* NBRC 108639^T^	*P. suffuscus* NBRC 105367^T^
2.2 min (1)	–	597.29 ^t^	–	–
2.5 min (2)	–	554.28 ^t^	–	–
2.8 min (3)	656.31 ^t^	–	656.31 ^t^	656.31 ^t^
6.3 min (4)	–	–	–	505.40 ^t, i^
7.2 min (5)	–	–	812.44 ^t, i^	–

–, not observed; ^t^ and ^i^, observed when cultured on double diluted TSA and double diluted ISP-2 agar, respectively.
